# Neuro-Modulating Effects of Honokiol: A Review

**DOI:** 10.3389/fneur.2013.00130

**Published:** 2013-09-11

**Authors:** Anna Woodbury, Shan Ping Yu, Ling Wei, Paul García

**Affiliations:** ^1^Department of Anesthesiology, Emory University, Atlanta, GA, USA; ^2^Department of Anesthesiology, Veteran Affairs Medical Center, Atlanta, GA, USA

**Keywords:** honokiol, neuroprotection, GABA, stroke, inflammatory pain, amyloid, magnolol, analgesia

## Abstract

Honokiol is a poly-phenolic compound that exerts neuroprotective properties through a variety of mechanisms. It has therapeutic potential in anxiety, pain, cerebrovascular injury, epilepsy, and cognitive disorders including Alzheimer’s disease. It has been traditionally used in medical practices throughout much of Southeast Asia, but has now become more widely studied due to its pleiotropic effects. Most current research regarding this compound has focused on its chemotherapeutic properties. However, it has the potential to be an effective neuroprotective agent as well. This review summarizes what is currently known regarding the mechanisms involved in the neuroprotective and anesthetic effects of this compound and identifies potential areas for further research.

## Introduction

Honokiol is a naturally occurring, pleiotropic lignan that can be extracted from *Magnolia grandiflora*, a species of magnolia common to Japan, and is used in traditional medicines throughout much of Asia. This compound has also been found in several other *Magnolia* species, including *Magnolia dealbata*, an endangered endemic species found in Mexico (1–3). Like the flavorful tannins present in wine, honokiol, and its structural analog, magnolol, are poly-phenolic compounds; both with a fragrant and spicy odor. Although aromaticity is not uncommon among anesthetics (lidocaine, propofol, etomidate, etc.) only propofol has a phenol ring like honokiol and magnolol. The structural homology likely explains some of the similar pharmacologic action (Table 1) (4, 5).

**Table 1:** **Structure and properties of phenolic compounds with neuroprotective bio-activity**.

	Propofol	Honokiol	Magnolol
Chemical structure			
	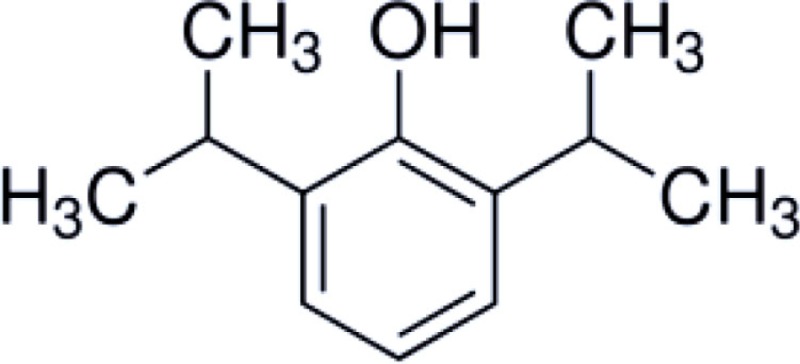	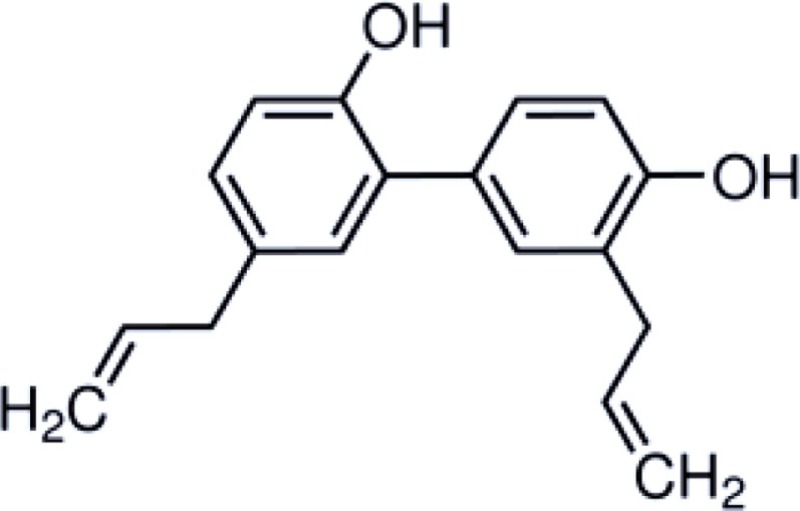	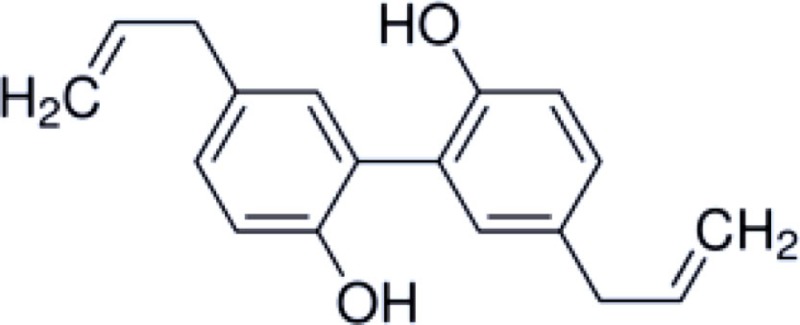
Solubility	Lipid	Lipid	Lipid
Receptors with known activity	GABA_A_, Na^+^ channel	GABA_A_, NFkB, NMDA	GABA_A_, NFkB, NMDA
Applications	Sedative-hypnotic, anti-emetic, neuroprotectant	Anxiolytic, analgesic, anti-inflammatory, anti-tumor, neuroprotectant	Antibacterial, antioxidant, anti-inflammatory, neuroprotectant

Honokiol is known for its anxiolytic (6–9), analgesic (10), antidepressant (11, 12), antithrombotic (13), antimicrobial (14–16), antispasmodic (17), anti-tumorigenic, and neuroprotective properties (18–27). While its pharmacokinetics have been studied in rodent models, these parameters have yet to be defined in humans (28). In rats, i.v. injection of honokiol 5–10 mg/kg has a plasma *t*_1/2_ of approximately 40–60 min (29). Meanwhile, intraperitoneal injection in mice of 250 mg/kg has yielded a *t*_1/2_ of 4–6 h, with a *t*_max_ of about 20–30 min (30). Due to its gaining popularity in western medical research, honokiol has been modified and synthesized for delivery through various modalities, including oral, intravenous, liposomal, and transdermal preparations (31–33). Honokiol may have some benefits in the peri-operative period, not only for its anxiolytic, analgesic, and antimicrobial effects, but also specifically for its potential role in neurologic and oncologic procedures. This review focuses primarily on honokiol’s effects in the central and peripheral nervous systems and its role in neuroprotection as related to its anxiolytic, analgesic, and anti-inflammatory actions.

## Neuroprotection

**Figure 1:**
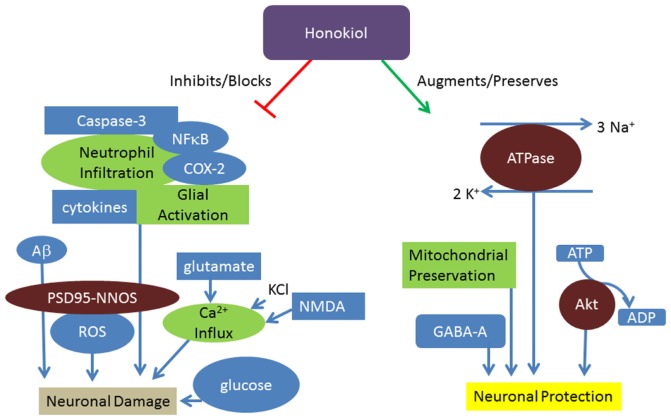
**Honokiol promotes neuroprotection through various mechanisms**. Honokiol results in neuronal protection through preservation of Na^+^/K^+^ ATPase, phosphorylation of pro-survival factors, preservation of mitochondria, and modulation of GABA_A_. Honokiol further promotes neuronal health through prevention of glucose, ROS, and inflammatory mediated damage.

It is known that honokiol can readily cross the blood brain barrier in order to exert its anti-tumorigenic effects in the central nervous system (34, 35). It might be assumed that because of its accessibility to neuronal tissue, honokiol has direct beneficial effects on cellular health as opposed to neuroprotection via promotion of alternate endogenous pathways. However, honokiol appears to exert its neuroprotective effects through a wide range of mechanisms (Figure 1).

In pre-clinical investigations, honokiol has been found to mitigate the effects of stroke and seizure and improve performance on learning and memory tests in behavioral models. Liou et al. administered honokiol intravenously either pre-ischemia [i.e., 15 min before unilateral middle cerebral artery (MCA) occlusion] or post-ischemia (i.e., administered upon removal of both common carotid arteries clips). Intravenous infusion of honokiol 10^−4^ and 10^−3^ mg/kg in both groups significantly reduced the total volume of infarction without creating significant hemodynamic changes (25). A subsequent study involved administration of intravenous honokiol 15 min before or 60 min after MCA occlusion leading to a dose-dependent reduction of the infarct volume by 20–70% (26). Similarly, in a rodent model of seizure disorder, the behavioral and neurotoxic effects of injection of NMDA into the cerebral ventricular system following acute seizure were measured by impairments in locomotion, climbing, and rotarod performance. These neurotoxic impairments were ameliorated by treatment with honokiol alone or combined with the NMDA antagonist, memantine. Tea polyphenol or memantine alone only partially ameliorated these behavioral impairments, indicating a comparatively augmented neuroprotective role for honokiol in seizure disorder (36).

Honokiol’s neuroprotective effects may be related to the promotion of healthy connections among nerve cells, as honokiol and its synthetic analogs have potent neurotrophic activity *in vitro* (37–39). Several putative pathways for neuroprotection have been investigated including inhibition of the immune system and oxidative stress pathways. For example, Chen et al. suggest that honokiol preserves Na^+^/K^+^-ATPase activity and enzymatic mitochondrial function in mice (19). Meanwhile, Harada et al. suggest that protection post-ischemia by honokiol can be attributed to suppressing the development of post-ischemic glucose intolerance and subsequent neuronal damage (21). In rats, Liou et al. found that honokiol also ameliorates cerebral infarction from ischemia-reperfusion injury via inhibition of neutrophil infiltration and reactive oxygen species (ROS) production as measured by luminol amplified chemiluminescence in neutrophils (25, 26). Another group examined the effects of honokiol following cerebral-ischemia-reperfusion injury, and determined that disruption of the postsynaptic density protein PSD95 and calcium-dependent neuronal nitric oxide synthase (nNOS) interface is the primary mechanism of honokiol’s protective effect, since PSD95-nNOS coupling to the NMDA receptor allows neurotoxic calcium influx through the NMDA receptor channels and could thereby result in neurotoxicity (23). Recently, inducers of the inflammatory cascade have been implicated in honokiol’s neuroprotective properties as well. Specifically, honokiol inhibits the inflammatory reaction during cerebral-ischemia-reperfusion by suppressing glial NFκB activation and cytokine production (27). Thus, it seems that honokiol may exert its neuroprotective effects during and after cerebral ischemic injury through a variety of mechanisms.

In addition to its potential role in stroke therapy, honokiol may have a role in seizure prevention as well. Honokiol’s central depressant activity was first described in the 1980s (40). Subsequently, both honokiol and magnolol have been evaluated as potential therapies for epilepsy and related disorders. Using rat cerebellar granule cells, Lin et al. observed the ability of honokiol to inhibit repetitive firing by blocking glutamate, NMDA and K^+^ evoked cationic influx. Other studies using intraperitoneal injections of honokiol or magnolol (1 and 5 mg/kg) were determined to increase seizure threshold with intravenous infusion of NMDA (10 mg/ml). Although both compounds significantly increased seizure thresholds, honokiol appeared to be more potent (41).

The potential for honokiol to promote cognitive health has been further investigated using neuronal cultures and animal models designed to test learning and memory. Oral honokiol (1 mg/kg) and magnolol (10 mg/kg) prevented the age-related memory and learning deficits found in senescence-accelerated mice by preserving cholinergic neurons and enhancing phosphorylation and activity of Akt, a member of the pro-survival pathway (42). Hoi et al. examined nine isolated compounds from Chinese herbs (honokiol, magnolol, vitamin E, salidroside, bilobalide, baicolin, gastrodin, α-asarone, and β-asarone) for potential anti-Alzheimer’s activity in neuronal cultures, but only honokiol and magnolol significantly decreased β-amyloid (Aβ)-induced neuronal death (22). Decreased ROS production, suppressed intracellular calcium elevation, and inhibition of caspase-3 activity may all contribute to honokiol’s neuroprotective effects in Aβ toxicity. The potential for honokiol to be therapeutic for neurodegenerative disease is supported by evidence that a honokiol-containing extract of *Magnolia officinalis* (10 mg/kg in 0.05% ethanol) prevents lipopolysaccharide (LPS)-induced memory deficiency via its antineuroinflammatory and antiamyloidogenic effects (43).

## Anxiolysis and Sleep

Honokiol’s central depressant effects may contribute not only to its anticonvulsant activity but also to its anxiolytic activity at low doses. This can be partially attributed to its interaction with the GABA_A_ receptor, a known target for benzodiazepines and other anxiolytics. When Kuribara et al. examined the effects of orally administered honokiol in an animal behavior model, they showed that a single oral dose of honokiol increased exploratory behavior while decreasing anxiety-related behavior. This effect was observed with repeated doses as low as 0.2 mg/kg. Honokiol achieved this with less motor or cognitive side effects than oral diazepam. This effect of honokiol was inhibited by administration of subcutaneous flumazenil, bicuculline (a GABA_A_ receptor antagonist), CCK-4, and caffeine. Flumazenil and bicuculline likewise disrupted the anxiolytic effect of diazepam, but CCK-4 and caffeine co-administration with diazepam had quite different outcomes. The combined administration of diazepam with caffeine enhanced diazepam’s anxiolytic effect, and diazepam completely reversed the effect of CCK-4. The authors concluded that honokiol produces anxiolysis with a better side effect profile than diazepam, and based on their results with caffeine, possibly through alternative pathways (9). Certain *ortho* (C2) and *para* (C4) substitutions increase the GABA-potentiating activity of phenols, and honokiol and magnolol have shown specific selectivity for different GABA_A_ receptor subtypes (44, 45). Taferner et al. examined modulation of GABA_A_ receptors by honokiol and derivatives as well, looking at subtype selectivity and structure-activity and finding a potential role of the acetamido group in subunit-dependent receptor modulation (46).

Glutamic acid decarboxylase (GAD) is an enzyme involved in GABA synthesis, and the activity of hippocampal GAD was significantly increased in honokiol-treated mice, suggesting that honokiol may alter the brain’s synthesis of GABA (47). Because GABA_A_ receptors have subunit heterogeneity that influences their function, Alexeev et al. explored the activity of magnolol and honokiol on neuronal and recombinant GABA_A_ receptors. Both compounds enhanced GABA-ergic neurotransmission in hippocampal dentate granule neurons. All recombinant receptors were sensitive to modulation, regardless of the subunit subtype, however, both compounds showed higher efficacy at receptors containing the δ subunit (900–1100% response with a tertiary δ subunit vs. 300–500% response with α, β, and γ subunits only) (48).

A recent study sought to explore the effect of honokiol on sleep. Honokiol (10 and 20 mg/kg) was found to significantly shorten sleep latency and to increase the amount of non-rapid eye movement sleep, though it had no effect on either the amount of rapid eye movement sleep or EEG power density. This group also found that honokiol excited sleep-promoting neurons in the ventrolateral preoptic areas (49). The sleep-promoting and anxiolytic effects of honokiol suggest a potential additional role for this neolignan in the anesthetic arena.

## Pain and Inflammation

Because of honokiol’s immunomodulatory effects, it is reasonable to assume that honokiol could be effective in inflammatory pain states. Formalin-induced paw licking is a common model for chronic inflammatory pain. Formalin produces a biphasic pain response following intraplantar injection, resulting in an acute pain phase that lasts approximately 5 min immediately following formalin injection, and an inflammatory pain phase that is observed 30–45 min following formalin injection, as evidenced by increased paw-licking behavior (50). Lin et al. showed that honokiol and magnolol reduced the inflammatory phase of formalin-induced paw-licking in mice and decreased formalin-induced c-Fos protein expression in superficial (I-II) laminae of the L4-L5 lumbar dorsal horn (51). This same group later studied the effects of honokiol and magnolol on paw-licking and thermal hyperalgesia induced by glutamate receptor agonists injected into the hind paws of mice. They found that honokiol and magnolol were similar in their ability to block the pain responses induced by glutamate, substance P, and PGE2, but honokiol was more selective than magnolol for inhibition of NMDA-induced licking behavioral and thermal hyperalgesia. For example, the same dose of either honokiol or magnolol (10 mg/kg) reduced glutamate-induced licking time and increased the latency to paw withdrawal from hot water in a test of thermal hyperalgesia. Additionally, intraplantar injection of honokiol or magnolol reduced total licking time and thermal hyperalgesia induction by NMDA in a dose-dependent fashion. Although in substance P – induced thermal hyperalgesia, magnolol was found to be slightly more effective than honokiol at lower doses, for PGE2-induced nociception and hyperalgesia, honokiol significantly decreased behavioral responses to pain at lower doses than magnolol. At physiologically relevant dosages, both compounds were able to significantly attenuate PGE2-induced thermal hyperalgesia up to 120 min following pain induction (10).

In an effort to probe deeper into honokiol’s anti-inflammatory effects, Chao et al. used LPS to induce inflammation and found that honokiol decreased tumor necrosis factor-alpha secretion in mouse macrophages following LPS. Additionally, honokiol inhibited nitric oxide expression in macrophages, protein kinase C-α membrane translocation, and NF-κB activation (52). This anti-inflammatory action can be translated to human monocyte-derived dendritic cells (53). These studies are supported by work from Murakami et al. who found similar inhibitory effects of honokiol on NF-κB activation and COX-2 expression (54).

Thus, it seems that honokiol’s analgesic effects are multifactorial, likely involving the inflammatory cascade, the NMDA receptor, and inhibition of well-known inflammatory pain mediators (e.g., glutamate and substance P). Honokiol would likely be a useful adjunct for treatment of inflammatory pain states, once its side effect profile has been adequately elucidated.

## Future Directions and Potential Risks

Honokiol appears to have several potential roles as a therapeutic for anesthesiologists, neurologists, and pain physicians. Although it has been used in traditional medicines for thousands of years, a thorough understanding of its bio-activity will aid in applying this compound or its derivatives to human pathologic conditions. It appears to specifically target tumorous cells in the CNS while preserving and protecting healthy neurons and preventing deleterious results from ischemia, seizure, amyloidosis, anxiety, depression, and pain (8, 9, 11, 12, 22, 28, 35, 55, 56). It is possible that many of honokiol’s downstream anti-inflammatory effects could result from modulation at a single upstream site that is yet to be elucidated. Its lipid solubility likely further contributes to its ability to act at multiple sites by allowing the compound to cross lipid bi-layers and exert its action intracellularly and beyond the blood brain barrier. Perhaps the reason for honokiol’s pleiotropic effects can be attributed partially to its chemical structure, which allows it certain similarities to propofol. Polyphenols, like phenols, are small compounds capable of interacting with several membrane proteins through hydrogen bonding, hydrophobic interactions, or the sharing of pi electrons due to aromaticity. Propofol, like honokiol, has a role not only as an anesthetic, but more recently as a neuroprotectant, anti-inflammatory, and anti-tumorigenic agent as well (57–59). It is likely that as further research is performed using propofol, honokiol, and other compounds with similar structure, more commonalities will be discovered among them, which will help to further define their mechanisms of action.

Honokiol is not entirely without risks, although its limited empirical application to humans at therapeutic doses has limited the evaluation of its side effect profile thus far. There are potential risks that can be expected, including increased bleeding and potential neurotoxicity at high doses. Honokiol has been found to be a potent inhibitor of arterial thrombosis, so it may be advisable to avoid honokiol in coagulopathic patients or in those where bleeding or hemorrhage may be of concern (13). These may include patients with hemorrhagic stroke, patients with hemorrhagic changes following ischemic stroke, patients on coumadin or therapeutic lovenox, and patients with clotting disorders such as hemophilia or von Willebrand’s deficiency. And, while honokiol has been found to have neuroprotective effects at low doses, it has also been found to increase neuronal death *in vitro* at higher doses (100 μM applied directly to fetal cortical neurons) (38). This suggests a need to further study its pharmacokinetic and pharmacodynamic parameters in humans before unintentionally administering a potentially toxic dose of this compound. Although the pharmacodynamics and pharmacokinetics of honokiol have now been well-studied in rats, further studies need to be performed in humans before it can be widely administered (29, 60, 61). With modifications of its structure and methods of delivery helping to improve its functionality at multiple targets, including the GABA receptor and its subunits, serotonergic receptors, and members of the inflammatory cascade, honokiol stands as a promising new therapeutic agent for a variety of conditions.

## Author Contributions

AW, researched references, wrote the paper, assembled figures/tables; SPY, wrote the paper, added/checked references, assisted in assembling figures/tables; LW, wrote the paper, added/checked references, assisted in assembling figures/tables; PSG, wrote the paper, added/checked references, assisted in assembling figures/tables.

## Conflict of Interest Statement

The authors declare that the research was conducted in the absence of any commercial or financial relationships that could be construed as a potential conflict of interest.
